# Effects of Zika Virus Strain and *Aedes* Mosquito Species on Vector Competence

**DOI:** 10.3201/eid2307.161633

**Published:** 2017-07

**Authors:** Alexander T. Ciota, Sean M. Bialosuknia, Steven D. Zink, Matthew Brecher, Dylan J. Ehrbar, Madeline N. Morrissette, Laura D. Kramer

**Affiliations:** New York State Department of Health, Slingerlands, New York, USA (A.T. Ciota, S.M. Bialosuknia, S.D. Zink, M. Brecher, D.J. Ehrbar, M.N. Morrissette, L.D. Kramer);; State University of New York at Albany School of Public Health, Albany, New York, USA (A.T. Ciota, L.D. Kramer)

**Keywords:** Zika virus, vector competence, Aedes albopictus, Aedes aegypti, mosquitoes, vector-borne infections, viruses

## Abstract

In the Western Hemisphere, Zika virus is thought to be transmitted primarily by *Aedes aegypti* mosquitoes. To determine the extent to which *Ae. albopictus* mosquitoes from the United States are capable of transmitting Zika virus and the influence of virus dose, virus strain, and mosquito species on vector competence, we evaluated multiple doses of representative Zika virus strains in *Ae. aegypti* and *Ae. albopictus* mosquitoes. Virus preparation (fresh vs. frozen) significantly affected virus infectivity in mosquitoes. We calculated 50% infectious doses to be 6.1–7.5 log_10_ PFU/mL; minimum infective dose was 4.2 log_10_ PFU/mL. *Ae. albopictus* mosquitoes were more susceptible to infection than *Ae. aegypti* mosquitoes, but transmission efficiency was higher for *Ae. aegypti* mosquitoes, indicating a transmission barrier in *Ae. albopictus* mosquitoes. Results suggest that, although Zika virus transmission is relatively inefficient overall and dependent on virus strain and mosquito species, *Ae. albopictus* mosquitoes could become major vectors in the Americas.

Zika virus (family *Flaviviridae,* genus *Flavivirus*) is the latest in a series of arboviruses to successfully invade the Americas; cases locally acquired in the Western Hemisphere were first identified in Brazil in May 2015 (*1*), and invasion subsequently expanded throughout Latin America and into the United States. To date, >460,000 suspected cases of autochthonous transmission have occurred in at least 45 Western countries (http://www.paho.org). Zika virus was first isolated in Uganda in 1947 (*2*) but was not implicated in a major epidemic until an explosive outbreak occurred on the island of Yap in Micronesia in 2007 (*3*). Subsequent outbreaks occurred in Cambodia in 2010, French Polynesia in 2013, and surrounding South Pacific islands in 2014 (*4*). Phylogenetic studies have suggested that the South Pacific islands are probably the source of the current outbreak in the Americas (*5*).

Although Zika virus infection had generally been thought to be asymptomatic or to result in a mild febrile illness (*6*), the 2013 outbreak marked the first time the virus was implicated as a causative agent of Guillain-Barré syndrome (*7*). It has also been confirmed that Zika virus can have teratogenic effects (*8*), causing a spectrum of neurologic problems, referred to as Zika congenital syndrome, in developing fetuses (*9*), particularly in women infected during their first trimester of pregnancy (*10*). Although the primary route of transmission is through blood feeding by an infected mosquito, efficient sexual transmission (*11*) and long-term persistence in male reproductive tissues and fluids have been well documented (*12*).

Epidemiologic and laboratory studies have implicated various *Aedes* spp. mosquitoes as Zika virus vectors (*3*,*13*–*16*). In the Americas, *Ae. aegypti* mosquitoes are the primary vector for Zika virus, as they are for dengue and chikungunya viruses (*17*). *Ae. albopictus* mosquitoes potentially act as a secondary or supplemental vector (*18*). In the laboratory, mosquitoes of both species have been shown to be efficient vectors (*14*,*18*–*20*). However, few Zika virus isolates have been obtained from mosquitoes in the Americas and few experiments have assessed competence with currently circulating strains and representative mosquito populations. In addition, because previous experimental studies generally used individual blood meal doses with virus titers rarely achieved in nature, the relationship between viremia levels and vector competence is largely uncharacterized, making determination of the duration and likelihood of host transmissibility difficult. 

We conducted comparative studies of recent Zika virus isolates from the Americas and an isolate from the 2010 outbreak in Cambodia (*21*). Genetic differences identified among these strains translated to modest variability in replicative kinetics in vitro in mosquito (C6/36) and mammalian (Vero) cells. In addition, we characterized the dose response for vector competence in *Ae. aegypti* mosquitoes and a recently colonized *Ae. albopictus* mosquito population from New York, USA. 

## Methods

### Viruses

The New York State Department of Health (NYSDOH) Arbovirus Laboratory isolated Zika virus HND (2016–19563, GenBank accession no. KX906952) from serum from a patient who had traveled to Honduras in early 2016. Amplification was obtained by inoculating 100 μL of serum into shell vials (ViroMed Laboratories, Burlington, NC, USA) confluent with Vero cells (ATCC, Manassas, VA, USA), followed by centrifugation at 700 × *g* for 40 min at 37°C and an additional 4 days of growth (*22*). Zika virus CAM (strain FSS130325, GenBank accession no. JN860885; kindly provided by C. Pager, State University of New York at Albany, NY, USA) was originally isolated in 2010 from human serum in Cambodia and passaged 3 times on Vero cell culture and 1 time on C6/36 cell culture. Zika virus PR (kindly provided by the Centers for Disease Control and Prevention, Fort Collins, CO, USA; strain PRCABC59, GenBank accession no. KU5012 15), used for preliminary experiments, was initially obtained from serum of a patient who had traveled to Puerto Rico in 2015 and was passaged 3 times on Vero cell culture and 1 time on C6/36 cell culture. Nucleotide and amino acid sequence alignments were created with Zika virus coding regions by using the MegAlign module of the DNAStar software package (http://www.dnastar.com).

### In Vitro Growth Kinetics

We inoculated confluent monolayers of Vero and C6/36 cells with Zika virus strains in duplicate at a multiplicity of infection of 0.01 PFU/cell. After a 1-hour absorption period at 37°C (Vero) or 28°C (C6/36), the inoculum was removed and cells were washed twice with appropriate maintenance media. Cultures were maintained in 6-well plates with 3 mL of maintenance media (Eagle minimum essential medium with 2% fetal bovine serum) and incubated at 37°C (Vero) or 28°C (C6/36). Samples of 100 μL supernatant were harvested at days 1–4 (Vero) or 1–7 (C6/36) after infection, diluted 1:10 in media containing 20% fetal bovine serum, and stored at −80°C. Titrations were performed in duplicate, by plaque assay on Vero cells (*23*); mean titers for each time point were calculated and compared by *t*-test. Growth kinetics were compared by using repeated measured analysis of variance (ANOVA) and Tukey post hoc tests (GraphPad Prism version 5.0; GraphPad Software, La Jolla, CA, USA).

### Experimental Infections and Mosquito Competence

*Ae. albopictus* mosquitoes (kindly provided by Illia Rochlin, Suffolk County Health Department, Yaphank, NY, USA) were originally collected in Suffolk County in 2014 and subsequently colonized in the NYSDOH Arbovirus Laboratory. F5–F7 female mosquitoes from New York were used for experimental feedings. *Ae. aegypti* mosquitoes used for preliminary experiments were collected by C. Mangudo in Salta, Argentina, in 2014 and initially colonized by V. Micieli and L.D. Kramer at the Centro de Estudios de Parasitología y Vectores (La Plata, Argentina) before being shipped to the NYSDOH Arbovirus Laboratory for maintenance. F4–F5 females from Argentina were used for experimental feedings. *Ae. aegypti* mosquitoes (kindly provided by G.D. Ebel, Colorado State University, Fort Collins, CO, USA) were originally collected in Poza Rica, Mexico. F7–F8 females from Mexico were used for experimental feedings. For preliminary blood feeding experiments, *Ae. aegypti* mosquitoes from Argentina were fed Zika virus PR stock virus diluted 1:1, 1:5, or 1:20 in defibrinated sheep blood (Colorado Serum Co., Denver, CO, USA) with 2.5% sucrose. For feedings with freshly propagated virus, supernatant from infected C6/36 cultures was harvested at 96 h after infection (multiplicity of infection ≈1.0) and diluted 1:1 with blood-sucrose mixture without freezing. Female mosquitoes, 4–7 days of age, were deprived of sucrose for 18–24 h and offered blood meal mixtures by use of a Hemotek membrane feeding system (Discovery Workshops, Acrington, UK) with a porcine sausage casing membrane. For all subsequent experiments assessing dose-dependent vector competence, similarly prepared fresh C6/36 cultures of Zika virus HND and Zika virus CAM were used to feed *Ae. aegypti* mosquitoes from Mexico and *Ae.* *albopictus* mosquitoes from New York. In addition to undiluted supernatant, 1:20, 1:400, and 1:8,000 dilutions were made in C6/36 maintenance media before being mixed with blood. 

For all blood feeding experiments, mosquitoes were sedated with CO_2_ after 1 h of feeding, and fully engorged mosquitoes were transferred to 0.6-L cartons and maintained at 27°C for experimental testing. Infection, dissemination, and transmission rates were determined as previously described (*24*) on day 14 or 21 after feeding. After the mosquitoes were sedated, the legs were removed from 12–30 mosquitoes and placed in 1 mL mosquito diluent (20% heat-inactivated fetal bovine serum in Dulbecco phosphate-buffered saline plus 50 μg/mL penicillin/streptomycin, 50 μg/mL gentamicin, and 2 μg/mL Fungizone [Sigma Aldrich, St. Louis, MO, USA]). For 30 minutes, mosquitoes were allowed to expectorate into capillary tubes containing ≈20 μL fetal bovine serum plus 50% sucrose (1:1), at which time the mixture was ejected into 250 μL mosquito diluent. Mosquito bodies were then placed in individual tubes with mosquito diluent. All samples were held at −80°C until tested. To test for infection, dissemination, and transmission, we processed and screened bodies, legs, and salivary secretions, respectively, by Zika virus–specific quantitative reverse transcription PCR (*25*). Zika virus body titers were calculated from standard curves based on infectious particle standards created from matched virus stocks. Data were analyzed by using GraphPad Prism version 4.0. Rates were compared by using Fisher exact tests, and dose dependence was evaluated and compared by using linear regression analyses.

## Results

### In Vitro Characterization

**Figure 1:**
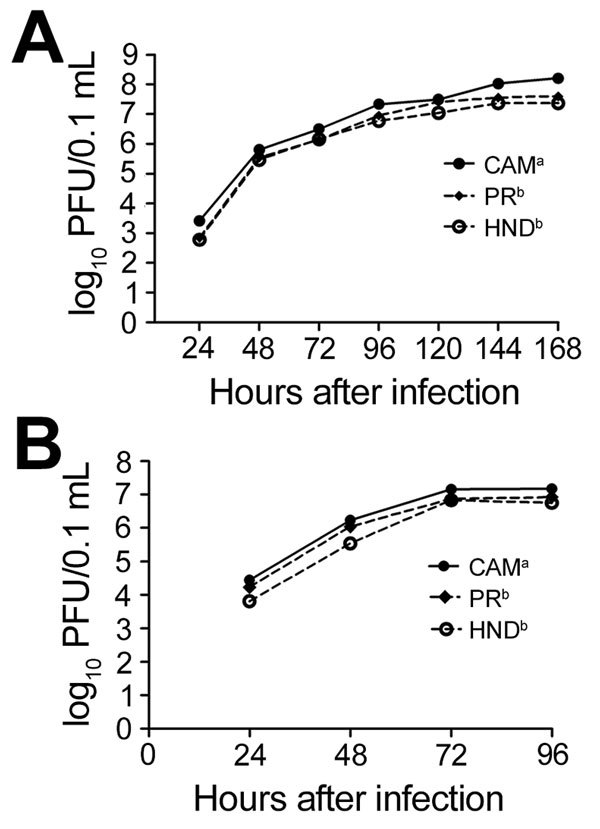
Growth kinetics of Zika virus in A) mosquito (C6/36) and B) mammalian (Vero) cells. Cells were infected in duplicate with Zika virus strain CAM, PR, or HND, at a multiplicity of infection of 0.1. Concentration of Zika virus in supernatant was determined by plaque titration for 4 (Vero) or 7 (C6/36) days after infection. Values represent geometric means ± SD, and different superscript letters represent statistically different growth kinetics (repeated measures analysis of variance; p<0.05 by Tukey post hoc test).

Sequencing analysis of these strains revealed 1.7% divergence and 16 aa differences distributed throughout the genome (Table 1). These differences include 8 aa in capsid; premembrane; envelope; and nonstructural 1, 3, and 5 genes, which differ from Zika virus CAM and are shared among the 2 isolates from the Americas. Peak virus titers were ≈3-fold higher on mosquito cells than on mammalian cells. Although in vitro kinetics were similar among strains (Figure 1), Zika virus CAM replicated to modestly higher titers (mean difference 3.0-fold by repeated measures ANOVA; p<0.05 by Tukey multiple comparison test) relative to Zika virus PR and HND and a significantly higher peak titer (mean 5.3-fold; p<0.05 by *t*-test). Zika virus HND was also modestly attenuated in mammalian cell culture, replicating to titers 2–3.3-fold lower than the titers achieved by Zika virus PR and Zika virus CAM, respectively (repeated measures ANOVA, by Tukey multiple comparison test). Peak titer for Zika virus CAM was statistically higher than that for Zika virus HND (p = 0.04 by *t*-test), yet Zika virus PR replicated to an intermediate value and was statistically equivalent to both Zika virus HND and CAM.

**Table 1:** Amino acid differences among Zika virus isolates used in study of species-specific Zika virus vector competence of *Aedes* mosquitoes*

Position	Gene	Zika virus strain, amino acids		
CAM	HND	PR
80	C	I	I	T
106	C	T	A	A
123	prM	V	A	A
130	prM	N	S	S
151	prM	M	L	L
620	E	V	V	L
763	E	V	M	M
894	NS1	G	A	G
982	NS1	A	V	V
1274	NS2A	P	L	L
1795	NS3	S	A	S
2074	NS3	M	L	M
2086	NS3	Y	H	H
2611	NS5	A	A	V
2634	NS5	M	V	V
3045	NS5	R	C	R

*C, capsid; E, envelope; NS, nonstructural; prM, premembrane.

### Infectivity and Vector Competence

**Figure 2:**
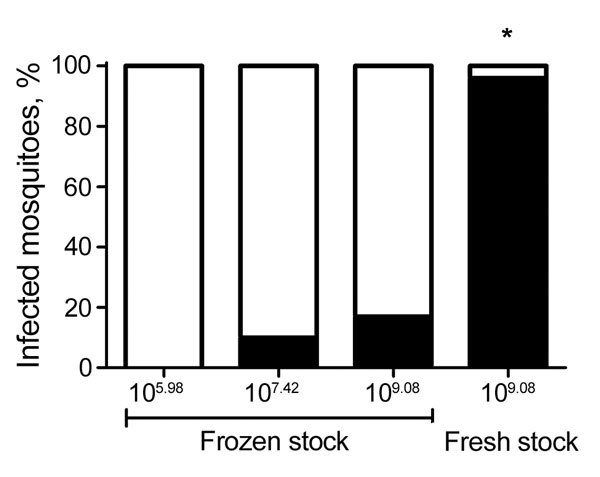
Relationship between dose, infectivity, and preparation of Zika virus for *Aedes aegypti* mosquitoes. Quantitative reverse transcription PCR was used to test 12–25 processed *Ae. aegypti* mosquitoes for Zika virus 14 days after exposure to infectious blood meals containing various doses of Zika virus PR. Frozen stocks had been stored at −80°C and thawed before blood meal preparation, and fresh stocks were used directly after propagation without freezing. The difference in proportion infected when fresh and frozen stock at equivalent titers were compared was highly significant. *p<0.0001 by Fisher exact test.

Initial experiments that used previously amplified Zika virus PR stock frozen at −80°C failed to achieve high levels of infection in *Ae. aegypti* mosquitoes. No infection was identified at 14 days after feeding for mosquitoes fed 6.0 log_10_ PFU/mL, and only 3 (10%) of 30 mosquitoes were Zika virus positive when the dose was increased to 7.4 log_10_ PFU/mL (Figure 2). In an effort to achieve higher infectivity, we freshly harvested supernatant from mosquito cells after virus propagation and immediately used it for blood meal preparation. Blood meal titers for this experiment were high, 9.1 log_10_ PFU/mL, as were rates of infection and dissemination. At day 14 after feeding, 24 (96%) of 25 mosquitoes were Zika virus positive. Of the 24 positive mosquitoes, 22 (91.6%) had disseminated infections and 13 (54.2%) had Zika virus–positive saliva. To clarify the extent to which differences in infectivity were the result of virus titer or preparation (freshly propagated vs. frozen virus stocks), we fed a subset of mosquitoes the same blood meal (titer 9.1 log_10_ PFU/mL) after freezing at −80°C for 2 weeks. Although feeding rates were poor and survival was low for this cohort (n = 12), only 2 of the mosquitoes surviving to day 14 after feeding were Zika virus positive, which equated to a significantly lower infection rate than that obtained with freshly propagated virus (p<0.001 by Fisher exact test; Figure 2). All subsequent experiments were therefore completed with C6/36-derived Zika virus–positive supernatant before freezing.

**Figure 3:**
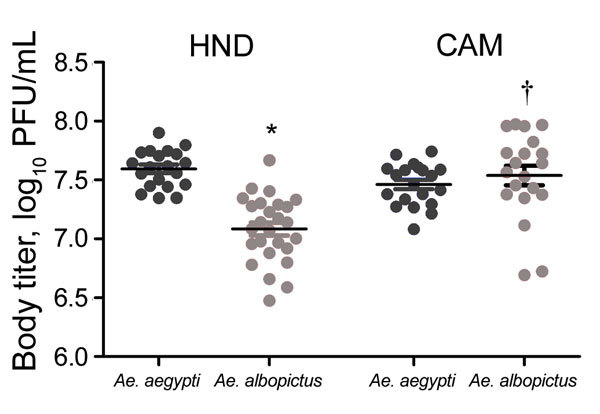
Viral load of Zika virus in *Aedes* mosquito bodies at day 21 after infection. Zika viral load (PFU equivalents) was determined in whole mosquitoes by using Zika virus–specific quantitative reverse transcription PCR and strain-specific standards. The graph shows titers in individual mosquitoes after feeding on the highest dose (8.6–8.9 log_10_ PFU/mL). Significant differences (*t*-test, p<0.05) were identified between mosquito species (*) and virus strains (†). Horizontal lines indicate means ± SD.

After isolation, Zika virus HND was used as the representative Western Hemisphere strain and compared with Zika virus CAM for all experiments assessing the relationships between dose, vector competence, virus strain, and mosquito species. To maximize transmission potential, we waited until day 21 after mosquito feeding to conduct these studies. For *Ae. aegypti* mosquito feedings, the highest doses achieved for Zika virus HND and CAM were 8.9 and 8.7 log_10_ PFU/mL, respectively. Similar titers of 8.9 (Zika virus HND) and 8.6 (Zika virus CAM) log_10_ PFU/mL were used for *Ae. albopictus* feedings (Table 2). For Zika virus HND, significantly higher viral loads were measured in *Ae. aegypti* relative to *Ae. albopictus* mosquitoes at both the highest dose and the 1:20 dilution (≈7.5 log_10_ PFU/mL; Figure 3; p<0.01 by *t*-test). Although differences were also measured at the lower doses, deviation is higher and statistical power is constrained by the smaller sample sizes. Viral loads among mosquitoes of each species were similar for Zika virus CAM, yet significantly higher than Zika virus HND in *Ae. albopictus* mosquitoes (Figure 3; p<0.001 by *t*-test), indicating an influence of mosquito species and of virus strain on Zika virus replication.

**Table 2:** Zika virus vector competence of *Aedes aegypti* and *Ae. albopictus* mosquitoes at 21 days after infection*

Mosquito species	Zika virus			Mosquitoes		
	Strain	Dose, log_10_ PFU/mL		Infected, % (no. tested)	Infected and disseminating, %	% Infected and transmitting
*Ae. aegypti*	HND	8.9		90.9 (22)	95.0	80.0†↑
		7.7		46.7 (30)†↓	85.7	78.0†↑
		6.6		16.7 (30)	40.0	40.0
		4.6		3.3 (30)	0	0
*Ae. aegypti*	CAM	8.7		80.0 (30)	100.0	75.0
		7.2		44.4 (26)	91.7†↑	75.0†↑
		5.6		10.0 (30)	66.7	33.3
		4.3		7.0 (30)	100	50.0
*Ae. albopictus*	HND	8.9		100.0 (30)	93.3	33.3S↓
		7.5		93.3 (30)†↑‡↑	75.0‡↑	21.4†↓
		5.9		33.3 (30)	40.0	10.0
		4.1		10.0 (30)	66.7	0
*Ae. albopictus*	CAM	8.6		95.2 (21)	95.0	55.0
		6.6		40.0 (30)‡↓	25.0†↓‡↓	25.0†↓
		5.3		23.3 (30)	85.7	14.3
		4.2		6.0 (16)	0	0

*Up and down arrows indicate value is significantly above or below the comparison value (p<0.05 by Fisher exact test). †Significant differences between different species at the same dose of the same virus strain. ‡Differences between viral strains at the same dose, within species.

Infection rates for high-dose feedings were 80%–100% (Table 2). Susceptibility, particularly for Zika virus HND, was generally higher among *Ae. albopictus* mosquitoes. This difference was highly significant for the Zika virus HND 1:20 dilution (≈7.5 log_10_ PFU/mL), for which 93.3% of *Ae. albopictus* mosquitoes were infected compared with 46.7% of *Ae. aegypti* mosquitoes (p<0.001 by Fisher exact test). Although the higher infection rate measured in *Ae. albopictus* mosquitoes was not significant at the 1:400 dilution (33.3% vs. 16.7%; p = 0.233 by Fisher exact test), it is notable that the input titer was ≈5-fold lower for the *Ae. albopictus* mosquito feeding (6.6 vs. 5.9 log_10_ PFU/mL), consistent with the increased infectiousness of Zika virus HND in this species.

**Figure 4:**
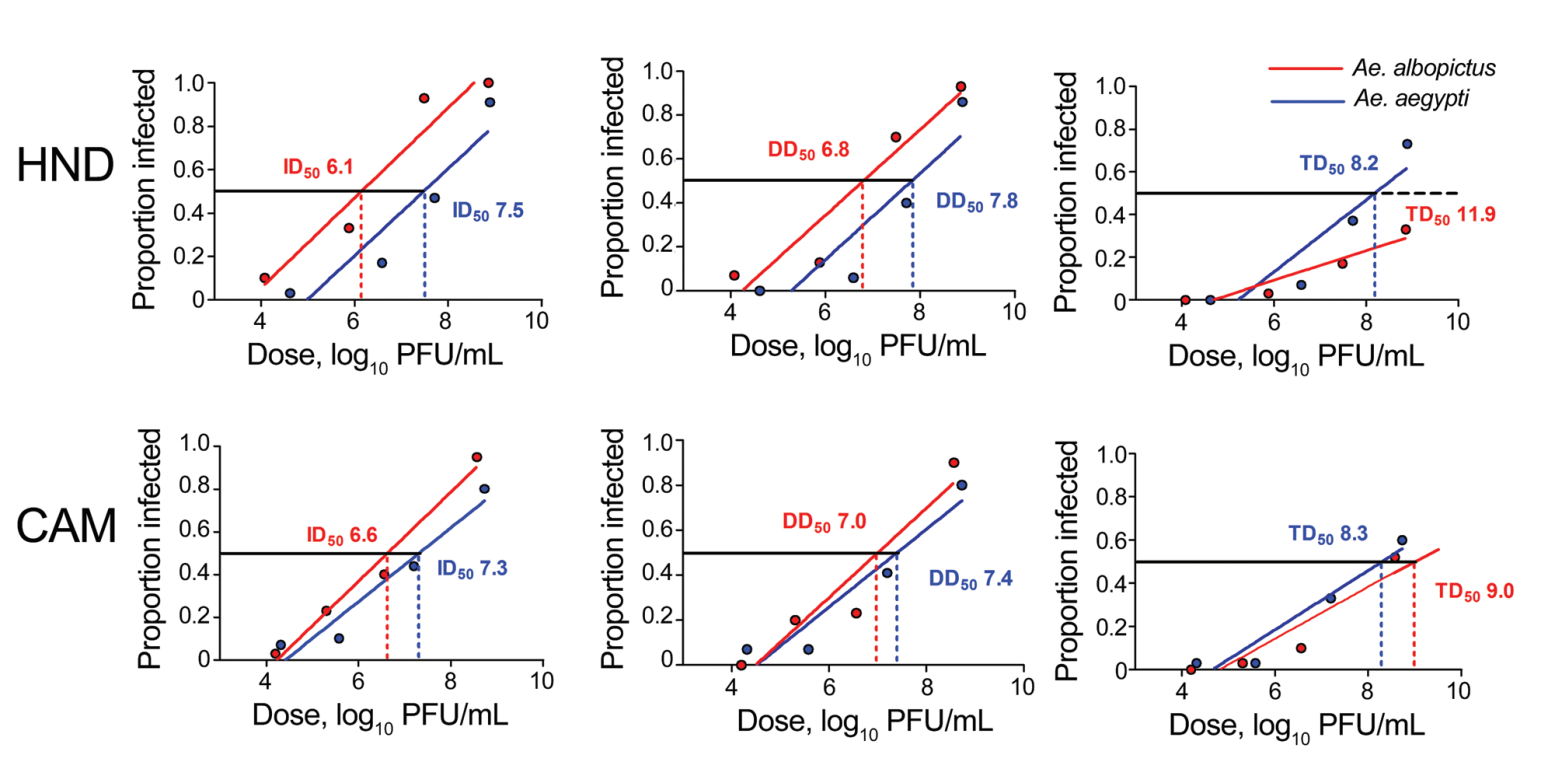
Relationship between dose and competence of *Aedes aegypti* and *Ae. albopictus* mosquitoes for Zika virus HND and CAM. Graphs show proportion of blood-engorged mosquitoes infected, with disseminated infections, and transmitting. Lines depict the best-fit linear relationships as determined by linear regression analyses. All relationships are linear and correlative (r^2^ = 0.82–0.97). Doses at which 50% of mosquitoes are infected, have disseminated infections, and are transmitting (ID_50_, DD_50,_ and TD_50_, respectively) were calculated by using best-fit lines.

To measure and compare dose dependence and competence among species and strains, we completed linear regression analyses of each individual feeding and used best-fit lines to calculate doses at which 50% of mosquitoes were infected, had disseminated infections, and were capable of transmission (ID_50,_ DD_50,_ TD_50,_ respectively; Figure 4). Although the relationship between dose and infection (as measured by slope) was similar among strains and species, infectiousness was higher in *Ae. albopictus* relative to *Ae. aegypti* mosquitoes for both strains. Infectiousness was determined both by statistical comparison of Y-intercepts by linear regression analyses (p<0.05) and comparisons of calculated ID_50s_. The ID_50_ of Zika virus HND was found to be greater than a log lower in *Ae. albopictus* compared with *Ae. aegypti* mosquitoes (6.1 vs. 7.5 log_10_ PFU/mL) and ≈5-fold lower for Zika virus CAM (6.6 vs. 7.3 log_10_ PFU/mL; Figure 4).

The increased infectivity of *Ae. albopictus* mosquitoes did not translate to increased rates of dissemination and transmission (Table 2; Figure 4) and is consistent with the fact that similar (Zika virus CAM) or lower (Zika virus HND) viral loads were measured in mosquitoes of this species (Figure 3). Dissemination rates for infected individuals were generally statistically equivalent among strains and species. Significant differences were achieved only when compared with the *Ae. albopictus* mosquito 1:20 Zika virus CAM feeding, yet the input titer for this feeding was ≈1 log_10_ lower than that for the other 1:20 feedings, which probably contributed to this difference. The linear regression analysis, for which the exact dose is considered, demonstrates again that the relationship of dissemination rate and dose is similar among virus strain and mosquito species (Figure 4). Although DD_50_ values for both strains were lower in *Ae. albopictus* mosquitoes, differences were significant only for Zika virus HND and can be wholly explained by differences in infectivity.

Transmission rates for *Ae. aegypti* mosquitoes were consistently higher, particularly for Zika virus HND (Table 2). Although no transmission occurred in mosquitoes of either species at the lowest dose, an average of 45% more Zika virus HND–infected *Ae. aegypti* than *Ae. albopictus* mosquitoes transmitted virus. These differences were highly significant at the undiluted and 1:20 doses (≈8.8 and 7.5 log_10_ PFU/mL; p<0.001 by Fisher exact test; Table 2). Although transmission of Zika virus CAM was also higher among *Ae. aegypti* mosquitoes, differences were smaller and only significant at the 1:20 dose (p<0.001 by Fisher exact test; Table 2). It is notable that the only instance for which transmission was measured at the lowest dose (1:8,000, 4.3 log_10_ PFU/mL) was with Zika virus CAM in *Ae. aegypti* mosquitoes. Despite increased infection rates for *Ae. albopictus* mosquitoes, dose dependence for Zika virus HND transmission was significantly lower than that for *Ae. aegypti* mosquitoes (p = 0.035 by linear regression analysis; Figure 4), resulting in a TD_50_ ≈4 log_10_ higher for *Ae. albopictus* mosquitoes. Overall transmission efficiency was highly strain dependent for *Ae. albopictus* mosquitoes; the TD_50_ of Zika virus CAM was ≈3 log_10_ lower than that for Zika virus HND. Unlike slopes and intercepts for Zika virus HND, those for Zika virus CAM TD_50_ were similar among species (Figure 4).

## Discussion

Reports of autochthonous Zika virus transmission in Florida demonstrate the capacity of Zika virus to continue to expand in the Americas (*26*), yet a comprehensive assessment of the current and future threat requires experimental assessment of the transmission potential of various mosquito populations and circulating strains. Our initial attempts to infect large numbers of *Ae. aegypti* mosquitoes by using previously frozen stocks of Zika virus were largely unsuccessful, even at unnaturally high doses (>9.0 log_10_ PFU/mL). Previous studies have noted differences in arbovirus infectivity and vector competence when use of artificial feeding was compared with feeding on experimentally infected hosts (*27*) or previously frozen to freshly propagated stocks (*28*–*30*). These studies almost exclusively identified significant differences in vector competence with lower titer blood meals, yet our results clearly demonstrate that freezing/thawing of Zika virus significantly impairs infectivity to mosquitoes at a range of doses. Although the mechanistic basis of this difference has not been adequately studied and plaque assays did not indicate a decline in Zika virus infectious particles on Vero cell culture after freeze/thaw, differences in competence may be attributed to structural perturbations of the virion that inhibit efficient particle binding in vivo (*31*). Future studies characterizing Zika virus structure and binding could help elucidate the unique sensitivity of this virus to the negative effects of freeze/thaw.

Although information about Zika virus kinetics and tropism in humans remains limited, current estimates of mean viremia range from 4.4 to 4.7 log_10_ copies/mL, probably equating to <2.5 log_10_ PFU/mL (*25*,*32*). Symptomatic persons may at times have higher levels of viremia (*32*,*33*), and these estimates are probably low because sample acquisition generally occurs after symptom onset (well past peak viremia) and because titers may be higher in whole blood than in serum (*34*). Despite these caveats, current data still suggest that peak and mean levels for dengue and chikungunya viruses are substantially higher than those for Zika virus (*32*). Of course, transmission efficiency of host to vector is dependent on both host viremia and vector susceptibility. Calculations of ID_50_ for dengue and chikungunya viruses in *Aedes* spp. mosquitoes are variable but have generally been estimated to be <10^5^ PFU/mL (*35*–*37*). We estimated Zika virus ID_50_ to be 6.1–7.5 log_10_ PFU/mL, with a low threshold for infection of 4.2 log_10_ PFU/mL. The recent success of Zika virus in the Western Hemisphere unequivocally demonstrates the capacity for widespread transmission, yet the combination of lower host viremia levels and mosquito susceptibility suggests that the intensity of vector-to-host transmission could be less efficient than has been observed with previous epidemics of dengue and chikungunya virus infection. It is feasible that efficient sexual transmission could supplement current levels of mosquito transmission (*38*) or that vertical transmission among particular mosquito populations could play a larger role than is documented for other flaviviruses (*39*–*41*). In addition, as demonstrated here and in previous studies (*20*), Zika virus vector competence can vary by virus strain and population, so particular vector/virus combinations may be more efficient at maintaining transmission. Highly variable vector competence that is specific for population and virus strain is well documented for other flaviviruses (*42*,*43*), and specific mosquito/virus genotype-by-genotype interactions have been well described in the *Aedes* mosquito/dengue virus system (*42*,*44*–*46*). Our analysis reveals 13 aa differences between the Zika virus 2010 CAM and 2016 HND strains and additional base changes that could be associated with phenotypically relevant changes to RNA structure. Although the CAM strain is ancestral to the America strains, it is notable that the 2 America strains used in this study (HND and PR) possess 8 aa differences. More comprehensive genetic studies demonstrate a range of mutations among strains currently circulating in the Western Hemisphere (*5*), all of which could feasibly translate to variability in virus fitness and vector competence.

We have demonstrated that US populations of *Ae. albopictus* mosquitoes exposed to a Zika virus strain currently circulating in the Americas are competent vectors that may be capable of maintaining virus transmission. Although these mosquitoes have been colonized for >1 year and may not be fully representative of current populations, this population was derived from a location (Suffolk County, NY) adjacent to New York City, which is among the largest centers for the movement of Zika virus–exposed travelers. Indeed, the highest number of confirmed travel-associated cases of Zika virus infection in the United States are reported from New York state (http://www.cdc.gov/zika/geo/united-states.html). The combination of the recent success of *Ae. albopictus* mosquitoes in the region (*47*) and the influx of viremic patients is probably what led to the first documented locally acquired case of dengue virus infection in the state (Suffolk County, 2013; http://diseasemaps.usgs.gov). Despite this potential vulnerability, transmission intensity is dependent on more than host and vector competence and it is the frequent and highly anthropophilic feeding of *Ae. aegypti* mosquitoes that often results in their relative success as vectors, even in the absence of high competence (*48*). In addition, the increased infectivity of Zika virus in *Ae. albopictus* mosquitoes does not translate to increased overall competence; transmission efficiency was significantly higher for *Ae. aegypti* mosquitoes. These data are consistent with a midgut escape barrier and, more significantly, with a salivary gland infection or transmission barrier. Although it is well documented that these barriers are capable of preventing transmission (*49*), increased infectivity in *Ae. albopictus* mosquitoes is notable because the increased infectivity we measured in *Ae. albopictus* mosquitoes translates to more opportunity for adaptive events that could increase fitness or competence over time. The potential epidemiologic consequences for adaptation to *Ae. albopictus* mosquitoes are well documented for chikungunya virus, for which primary and secondary epistatic mutations increased competence in mosquitoes of this species and facilitated the explosive outbreaks in the islands of the Indian Ocean and beyond (*50*). The combination of additional laboratory studies assessing the adaptive potential of Zika virus in various *Ae. albopictus* mosquito populations, together with continued genetic and phenotypic monitoring of circulating strains, will help elucidate the potential for similar adaptive events enabling increased transmission efficiency of Zika virus in the Americas.
